# Effect of Intramuscular Atropine Sulphate and Glycopyrrolate on Heart Rate and Salivary Secretion in Patients Undergoing Minor Oral Surgical Procedure

**DOI:** 10.7759/cureus.11780

**Published:** 2020-11-30

**Authors:** P B Rachana, Joyce Sequeira

**Affiliations:** 1 Oral and Maxillofacial Surgery, Kurunji Venkatramana Gowda Dental College and Hospital, Sullia, IND; 2 Oral and Maxillofacial Surgery, Yenepoya Dental College, Mangalore, IND

**Keywords:** atropine sulphate, glycopyrrolate, salivary secretion, heart rate, intramuscularly, minor oral surgical procedure

## Abstract

Introduction

In most of the oral surgical procedures performed under local anesthesia, we often face a great difficulty while performing surgeries because of reduced accessibility and visibility which is hampered by blood and saliva at the surgical site. Anticholinergic drugs like atropine sulphate and glycopyrrolate are commonly used as antisialogogue for patients undergoing a surgical procedure under general anesthesia with little or no side effects.

Aims and objectives

To evaluate and compare the antisialogogue effect of atropine sulphate and glycopyrrolate in patients undergoing minor oral surgical procedures. To compare the efficacy of these drugs when administered intramuscularly and to evaluate their effects on heart rate in patients undergoing minor oral surgical procedures.

Materials and methods

Thirty patients undergoing minor oral surgical procedure were selected for the study. The patients were randomly assigned to receive either 0.6 mg/ml of Atropine Sulphate or 0.2 mg/ml of Glycopyrrolate intramuscularly. Salivary secretion, heart rate and arterial pressure were noted pre-injection and 30 minutes after the administration of the drug.

Results

Atropine sulphate and glycopyrrolate were equally potent as an antisialogogue. There was a significant increase in heart rate 30 min after the administration of atropine sulphate, but there was no significant change in heart rate in glycopyrrolate group.

Conclusion

Intramuscular gycopyrrolate is safer than intramuscular atropine sulphate as an antisialogogue in minor oral surgical procedures under local anesthesia.

## Introduction

The accessibility and visibility while performing surgical procedures in oral cavity are hampered by the collection of saliva and blood at the surgical site. Saliva hinders the visibility and thereby increasing the operating time and decreasing the efficiency of the surgeon. Saliva also contaminates the operating field with oral microbial flora. Cotton rolls, saliva ejectors, tongue retractors and high-volume suctions can be used to keep the operating field as dry as possible. Control of salivary flow may be desirable in periodontal and oral surgical procedure to improve the field of vision and to minimize the dehydration of patient during lengthy treatment demanding suctioning. There are several ways to block or reduce salivary flow including the use of botulinum toxin injection [1] and rerouting of the submandibular ducts [2], these techniques however primarily are long-lasting treatments for patients who experience uncontrollable drooling. Antisialogogues can be used for short, temporary reduction of saliva. For oral surgical procedures performed under general anaesthesia, antisialogogue is commonly administered often as a premedication and does provide effective drying.

Our study was instigated to compare the antisialogogue effect of atropine sulphate and glycopyrrolate and convenience of administration of these drugs with regard to minor oral surgical procedure performed under local anaesthesia.

## Materials and methods

After obtaining ethical clearance from the university ethics committee, randomized prospective study was conducted on 30 ASA grade 1 patients, age group ranged from 15 to 45 years who were undergoing minor oral surgical procedures like impaction, frenectomy, alveoloplasty, surgical removal of impacted teeth under local anesthesia. Patients who had undergone irradiated or with habits of pan chewing, smoking etc were excluded from the study. All the patients who were included in the study were given information regarding the procedure and informed consent was obtained. The patients were randomly divided into two groups. Patients were allocated to Group A and Group B in which an odd number of patients were included in Group A, and even number of patients were included in Group B. The sample size was 30 with 15 in each group.

Prior to the treatment, patient data was recorded as per the proforma followed by a clinical, radiographic and physical examination of the patient. Prior to administering glycopyrrolate and atropine, heart rate and arterial pressure were noted and salivary secretion was weighed using preweighed test tube fitted with a funnel. Commercially available 0.6mg of atropine sulphate (1 ampule) was administered intramuscularly into gluteal muscle to Group A and 0.2mg of glycopyrrolate (1 ampule) was administered intramuscularly into gluteal muscle to Group B. Thirty minutes after the injection, the parameters were noted again and surgical procedure was performed under local anaesthesia.

Method of collection of saliva

The patients were seated comfortably on the dental chair. Patients were asked to rinse with a plain glass of water to cleanse the teeth of loosely adherent debris. The samples of mixed saliva were collected in a preweighed test tube fitted with a funnel without artificial stimulation, for one minute.

## Results

Paired “t” test was used for the statistical analysis. The value of the parameters, comparison between the two groups and result of the study are summarized in Table 1 and Table 2. Results of our study showed that there was a significant decrease in salivary secretions in both the groups. During the course of the study, it was observed that there was significant increase in heart rate 30 min after administration of Atropine but there were no significant changes in heart rate 30 min after the administration of glycopyrrolate. There was no significant variation in arterial pressure in both the groups.

**Table 1:** Intra-group comparison

Parameters		Paired difference		t value	P value	Inference
		Mean	SD			
Atropine	Saliva (pre – post)	0.94000	0.38508	9.454	<0.001	Significant
	Heart rate (pre – post)	-16.0000	14.96186	-4.142	<0.001	Significant
	Systolic BP (pre – post)	-4.6667	6.39940	-2.824	0.014	Not significant
	Diastolic BP (pre- post)	-1.2000	8.06403	-0.576	0.574	Not significant
Glycopyrrolate	Saliva (pre – post)	1.0667	0.41173	10.034	<0.001	Not significant
	Heart rate (pre – post)	2.2000	5.51880	1.544	0.145	Not significant
	Systolic BP (pre – post)	0.6667	2.58199	1.000	0.334	Not significant
	Diastolic BP (pre- post)	-1.2000	3.18927	-1.457	0.167	Not significant

**Table 2:** Comparison between atropine and glycopyrrolate (inter-group)

Parameters		Atropine	Glycopyrrolate	t value	P value	Inference
Saliva		0.940±0.3858	1.066±0.4117	0.87000	0.392	Not significant
BP	Systolic	-4.6667±6.39940	0.6667±2.58199	2.99300	0.003	Significant
	Diastolic	-1.2000±8.06403	-1.2000±3.18927	0.000	1	Not significant
Heart rate		-16.000±14.962	2.200±5.519	4.42000	<0.001	Significant

## Discussion

Atropine and glycopyrrolate have muscarinic blocking (vagolytic) effects but exert no activity at the nicotinic receptors (neuromuscular junction). Evidence for subclasses of muscarinic receptors can be found in variable sensitivity among different muscarinic cholinergic receptors (muscarinic cholinergic receptors that control salivation are inhibited by lower doses of anticholinergic drugs than are required to inhibit muscarinic receptors at the heart and eyes) as well as in the difference in potency among the anticholinergic drugs. Oral absorption of anticholinergic drugs is not sufficiently predictable to warrant the use of this approach, absorption after intramuscular approach is prompt. Atropine and Scopolamine are lipid-soluble tertiary amine (easily penetrating the blood-brain barrier) whereas Glycopyrrolate is poorly lipid-soluble quaternary ammonium compound. Required intramuscular dose of atropine for reliable antisialogogue effect ranges from 10-20 microgram/kg body weight. Oral dryness and blurred vision are seen within 10 to 15 minutes after intramuscular administration of 0.4 to 0.6 mg of Atropine. Required intramuscular dose of glycopyrrolate for reliable antisialogogue effect is 5-8 microgram/kg body weight. The dosage used in our study were based on the study conducted by Mirakhur et al. and McCubin et al. [3,4].

Anticholinergic drugs placed topically on the cornea block the action of acetylcholine on the circular muscles of the iris (mydriasis and ciliary muscles). These changes could increase intraocular pressure in patients with glaucoma. Doses of intramuscular Atropine used for preoperative medication are probably inadequate to increase intraocular pressure even in susceptible patients assuming that medications used to treat glaucoma are continued. Anticholinergic causes bronchodilation and a decrease in bronchial secretion, respiratory rate may be increased. In therapeutic dose, mild vagal excitatory effects are noticed. In higher doses, central excitation is prominent manifesting as restlessness, delirium, disorientation and hallucination. This is followed by generalized CNS depression. Anticholinergic reduces the tone of lower oesophageal sphincter, salivary and gastric secretions are inhibited and gastric emptying is delayed. The normal papillary reflex is abolished. Physostigmine, 15 to 60 µg/kg intravenously is the specific treatment for anticholinergic drug overdosage (repeated doses of this anticholinesterase drug may be necessary to prevent recurrence of symptoms).

Bernstein et al. [5] found that oral administration of glycopyrrolate had limited role in salivary and gastric secretion, but intramuscular Glycopyrrolate significantly decreased salivary secretion, but did not affect gastric secretion. In his study intravenous administration of Glycopyrrolate on the other hand significantly decreased both salivary and gastric secretion because of higher peak level in plasma which was not achieved by intramuscular route. In our study 0.2 mg glycopyrrolate and 0.6 mg Atropine Sulphate when administered intramuscularly showed significant reduction in salivary secretion 30 min after administration. Results of our study showed that there was a significant decrease in salivary secretions in both the groups. As an antisialogogue for minor oral surgery, there was no significant difference in the potency of atropine and glycopyrrolate. Our study result is concurrent with the result of the study conducted by Gronnebech et al. [6].

In our study, 0.2mg administration of Glycopyrrolate intramuscularly did not show any significant increase in heart rate. This difference can be explained by understanding of the nervous innervations. Heart rate is vagally regulated as is gastric secretion. Antisialogogue effect are mediated by glossopharyngeal and facial nerve, which is inhibited with much lower concentration of glycopyrrolate than is the parasympathetic function of the vagus nerve. During the course of our study it was observed that there was significant increase in heart rate 30 min after the administration of the Atropine Sulphate. However, there was no significant increase in the heart rate 30 min after the administration of glycopyrrolate. Our study was concurrent with the result of the study conducted by Greenan et al., Mirakhur et al. and Mostafa et al. [7-9].

There were no significant variations in arterial pressure in both the groups [10].

Our study has shown the relative effects of glycopyrrolate and atropine sulphate when administered intramuscularly, the glycopyrrolate produced a more stable heart rate with reduced frequency of tachycardia and arrhythmia. Being a quaternary ammonium compound, Glycopyrrolate crosses the blood-brain barrier to a minimal degree compared to Atropine Sulphate. Atropine cannot be administered to patients with cardiovascular problems. Glycopyrrolate is superior to atropine sulphate as an antisialogogue with stable cardiac rate.

## Conclusions

Intramuscular glycopyrrolate is safer than intramuscular atropine Sulphate as an antisialogogue with stable cardiac rate and rhythm with minimal side effects. However further studies on larger sample size are required for a definite conclusion.
